# Supplementary material for: Three-Dimensional Architecture of the Human BRCA1-A Histone Deubiquitinase Core Complex
Source: Cell Rep. 2016 Dec 22;17(12):3099–106. doi: 10.1016/j.celrep.2016.11.063 (PMC5199339; doi:10.1016/j.celrep.2016.11.063)
Supplement: Document S1. Supplemental Experimental Procedures and Figures S1–S3 [file mmc1.pdf]

Cell Reports, Volume *17*

Supplemental Information

Three-Dimensional Architecture of the Human BRCA1-A Histone Deubiquitinase Core Complex

Otto J.P. Kyrieleis, Pauline B. McIntosh, Sarah R. Webb, Lesley J. Calder, Janette Lloyd, Nisha A. Patel, Stephen R. Martin, Carol V. Robinson, Peter B. Rosenthal, and Stephen J. Smerdon

Supplemental Information

Three-dimensional architecture of the human BRCA1-A histone deubiquitinase core complex

Otto J.P. Kyrieleis, Pauline B. McIntosh, Sarah R. Webb, Lesley J. Calder, Janette Lloyd, Nisha A. Patel, Stephen R. Martin, Carol V. Robinson, Peter B. Rosenthal and Stephen J. Smerdon

Supplementary Experimental Procedures

Supplementary Figures S1, S2 & S3

Supplementary References

Supplementary Experimental Procedures

DUB activity assay

The DUB activity assay was carried out in 40 mM Tris pH=8.0, 100 mM NaCl, 1 mM TCEP. The active BRCA1 A complex core was combined with K63-linked penta-ubiquitin in a total volume of 15 µl and incubated at 37°C for 5, 10, 20, 30, 40, 50, 60, 90 and 120 min. The reaction was quenched by adding SDS sample buffer and by heating the sample to 100°C for 5 min. Samples were analysed by SDS-PAGE. The following enzyme concentrations were used in this DUB assay: [UB_5, K63-linked] =23.5 µM and [BRCA1-A] = 16.8 nM.

Tilt-pair analysis and determination of absolute hand

Tilt-pair images, at 20° and -20°, were obtained for the cross linked structure using the same grids and conditions as described above. Particle pairs were picked from tilted images using Xmipp tilt-pair picking function. Euler angles were assigned to both tilted and untilted particle stacks using 1 cycle of Frealign in mode 3 (global search) with the final map as a reference. Tilt-pair analysis (Rosenthal & Henderson, 2003) was performed using the tilt-service (Wasilewski and Rosenthal, 2014). The minimum in the tilt-pair parameter plot agreed with that expected for the known tilt-axis and tilt-angle used in the experiment confirmed by the presence of the expected defocus gradient in the image fields. However, the minimum was at a smaller angle than used in the experiment due to flattening of the particles in stain.

Figure S1

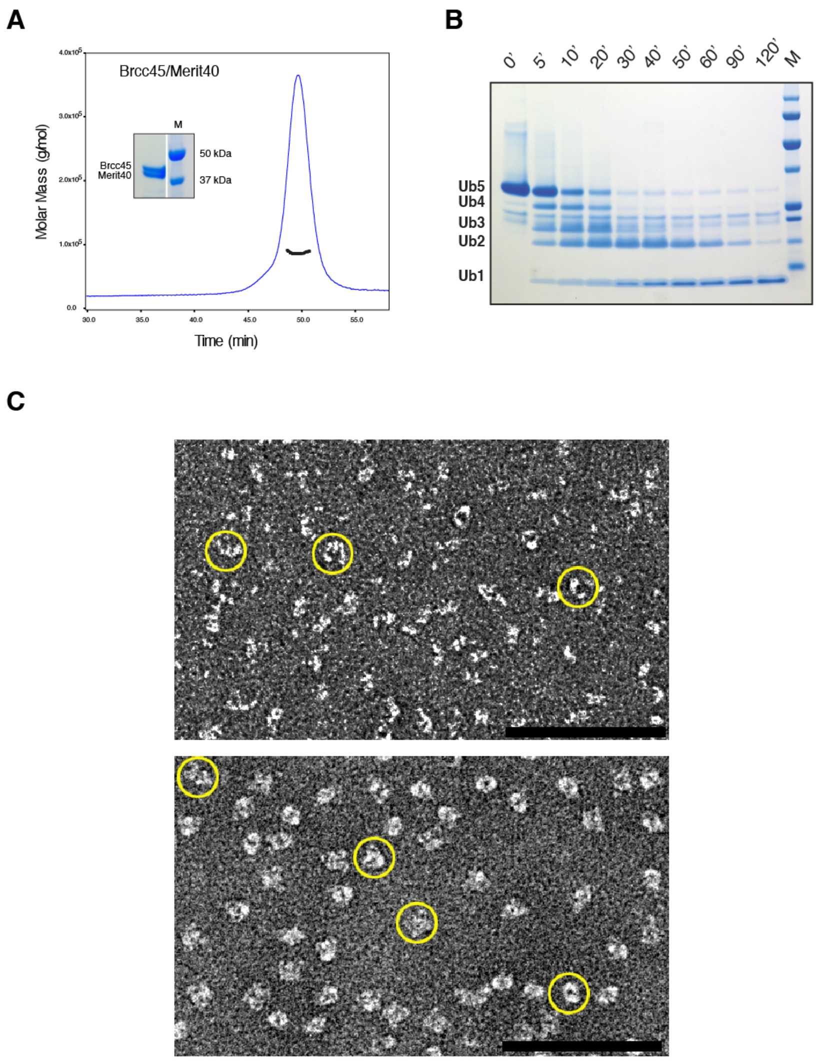

Figure S1 (Related to Figure 1) **BRCA1-A complex (A)** SEC-MALLS and SDS-PAGE analysis (inset) analysis of the purified BRCC45/MERIT40 complex. UV absorbance at 280 nm is shown in blue and the weight-averaged molecular mass across the peak in black. **(B)** Deubiquitinase assay of the BRCA1-A core complex with K63-linked penta-ubiquitin substrate analysed by SDS-PAGE. **(C)** Representative fields of raw negatively stained electron micrographs obtained from both un-crosslinked (Top) and crosslinked (Bottom) BRCA1-A complex, scale bars 100 nm. Representative views are circled.

Figure S2

A

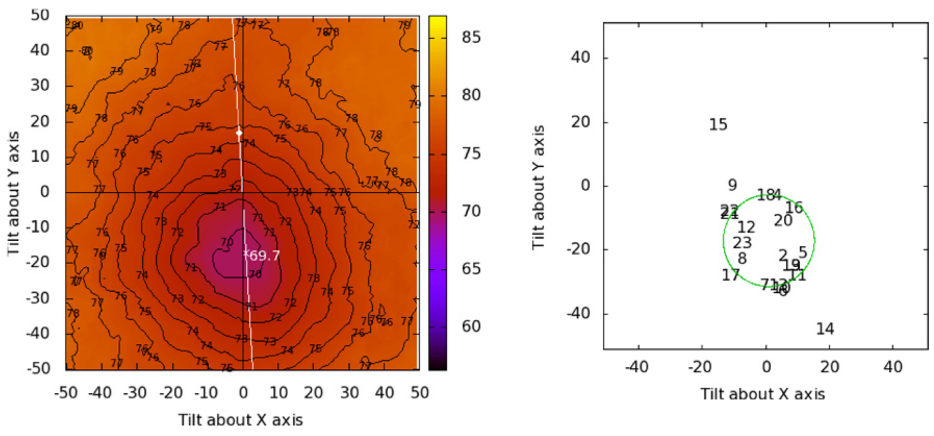

B

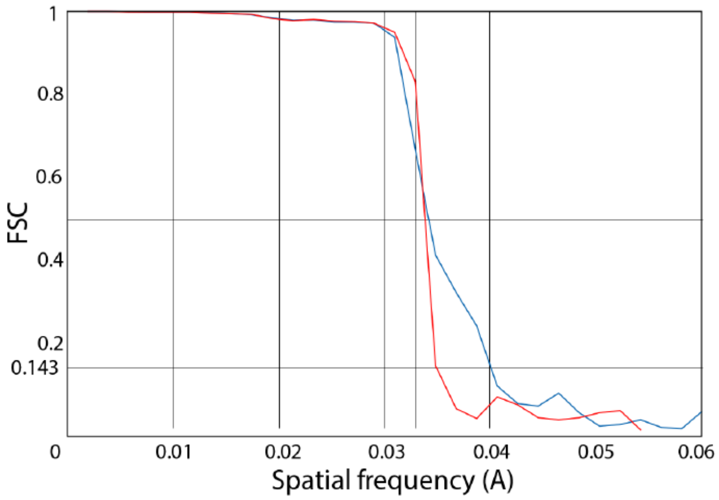

Figure S2 (Related to Figure 2) **EM analysis (A)** (Left) Tilt-pair phase residual plot and (Right) tilt-pair parameter plot shows a minimum and cluster respectively near the tilt-axis and tilt-angle (20 deg) used in the experiment. **(B)** Fourier–Shell Correlation for the crosslinked BRCA1-A complex between two maps calculated from different subsets of particle images (blue) and for refinement performed with phases randomized after 30A (red).

Figure S3

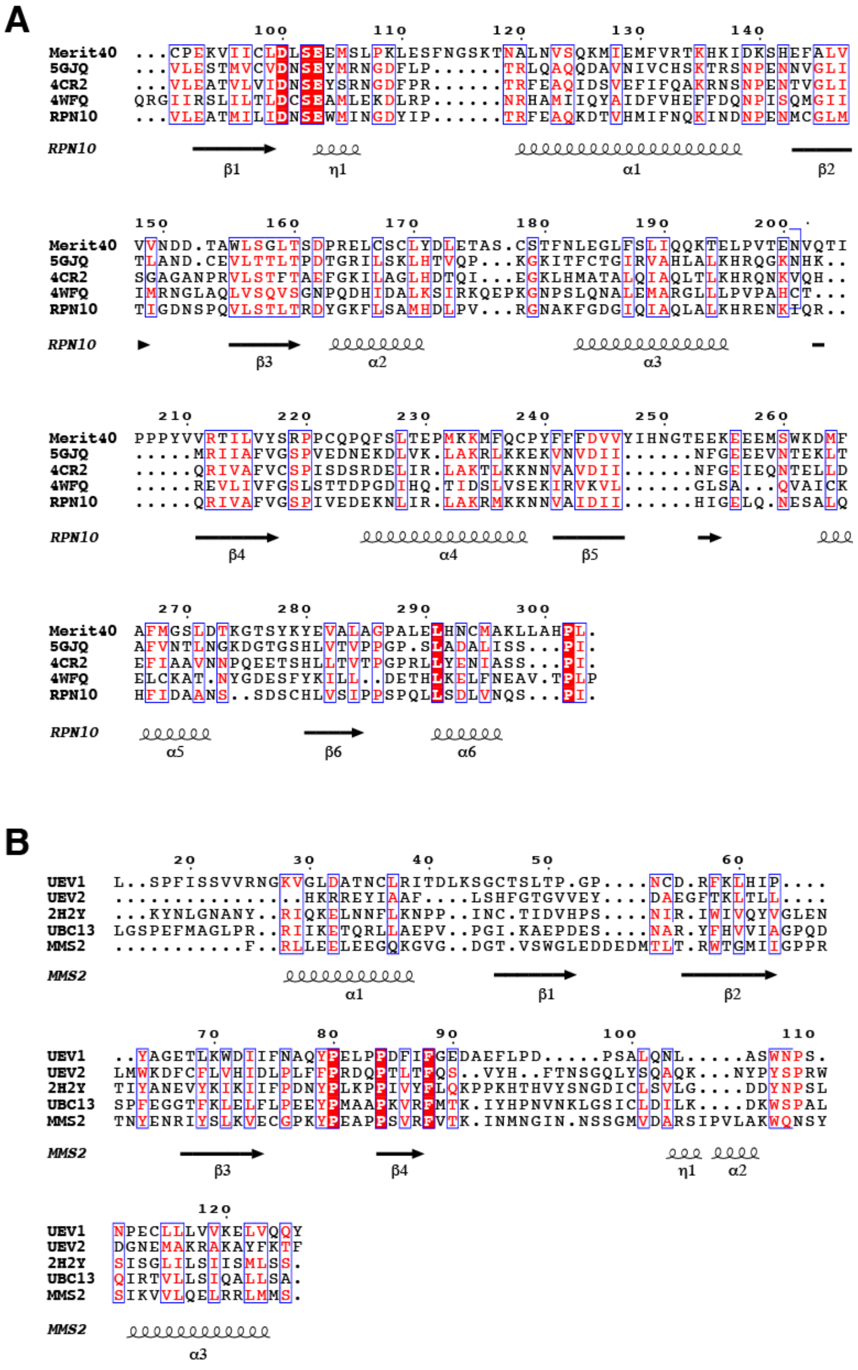

Figure S3 (Related to Figure 3) **Sequence homology** - Sequence alignments of the UEV domains of BRCC45 **(A)** and the VWA domain of MERIT40 **(B)** with homologous domains of known structure.

Supplementary References

Wasilewski S, Rosenthal PB Web server for tilt-pair validation of single particle maps from electron cryomicroscopy. *J Struct Biol* 2014, 186:122-131.

Rosenthal PB, Henderson R Optimal determination of particle orientation, absolute hand, and contrast loss in single-particle electron cryomicroscopy. *J Mol Biol* 2003, 333:721-745.
