# Supplementary material for: Three-Dimensional Architecture of the Human BRCA1-A Histone Deubiquitinase Core Complex
Source: Cell Rep. 2016 Dec 22;17(12):3099–106. doi: 10.1016/j.celrep.2016.11.063 (PMC5199339; doi:10.1016/j.celrep.2016.11.063)
Supplement: Document S2. Article plus Supplemental Information [file mmc2.pdf]

Cell Reports

Report

Three-Dimensional Architecture of the Human BRCA1-A Histone Deubiquitinase Core Complex

Graphical Abstract

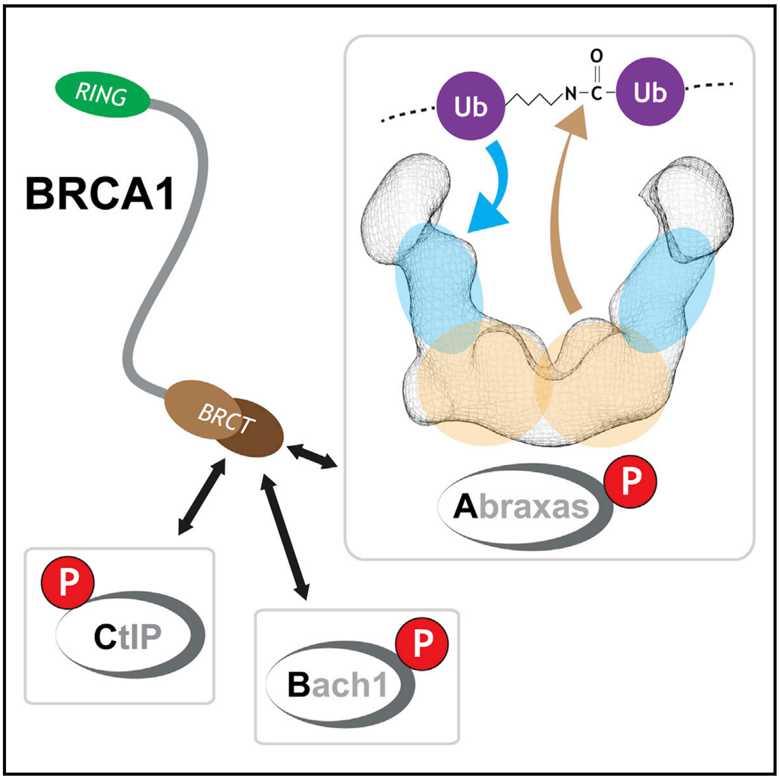

Highlights

- Structure of the core complex of the human BRCA1-A deubiquitinase
- V-shaped architecture likely conserved in the cytoplasmic BRISC complex
- BRCC45/BRE contributes to Lys63-linked ubiquitin chain binding

Authors

Otto J.P. Kyrieleis, Pauline B. McIntosh, Sarah R. Webb, ..., Carol V. Robinson, Peter B. Rosenthal, Stephen J. Smerdon

Correspondence

peter.rosenthal@crick.ac.uk (P.B.R.),
steve.smerdon@crick.ac.uk (S.J.S.)

In Brief

Kyrieleis et al. have used negative stain electron microscopy along with mass spectrometry and binding assays to characterize one of the major BRCA1-interacting complexes, BRCA1-A, showing the overall architecture of the core four-protein assembly and how this relates to its role as a histone deubiquitinase in double-stranded DNA break signaling.

Kyrieleis et al., 2016, Cell Reports *17*, 3099–3106
December 20, 2016 © 2016 The Author(s).
http://dx.doi.org/10.1016/j.celrep.2016.11.063

Cell Reports
Report

OPEN ACCESS
CellPress

Three-Dimensional Architecture of the Human BRCA1-A Histone Deubiquitinase Core Complex

Otto J.P. Kyrieleis,[1,5] Pauline B. McIntosh,[2,5] Sarah R. Webb,[1] Lesley J. Calder,[2] Janette Lloyd,[1] Nisha A. Patel,[4] Stephen R. Martin,[3] Carol V. Robinson,[4] Peter B. Rosenthal,[2,*] and Stephen J. Smerdon[1,6,*]
[1]Structural Biology of DNA-Damage Signalling Laboratory
[2]Structural Biology of Cells and Viruses Laboratory
[3]Structural Biology Technology Platform
The Francis Crick Institute, 1 Midland Road, London NW1 1AT, UK
[4]Department of Chemistry, Physical and Theoretical Chemistry Laboratory, University of Oxford, South Parks Road, OX1 3QZ Oxford, UK
[5]Co-first author
[6]Lead Contact
*Correspondence: peter.rosenthal@crick.ac.uk (P.B.R.), steve.smerdon@crick.ac.uk (S.J.S.)
http://dx.doi.org/10.1016/j.celrep.2016.11.063

SUMMARY

BRCA1 is a tumor suppressor found to be mutated in hereditary breast and ovarian cancer and plays key roles in the maintenance of genomic stability by homologous recombination repair. It is recruited to damaged chromatin as a component of the BRCA1-A deubiquitinase, which cleaves K63-linked ubiquitin chains attached to histone H2A and H2AX. BRCA1-A contributes to checkpoint regulation, repair pathway choice, and HR repair efficiency through molecular mechanisms that remain largely obscure. The structure of an active core complex comprising two Abraxas/BRCC36/BRCC45/MERIT40 tetramers determined by negative-stain electron microscopy (EM) reveals a distorted V-shape architecture in which a dimer of Abraxas/BRCC36 heterodimers sits at the base, with BRCC45/Merit40 pairs occupying each arm. The location and ubiquitin-binding activity of BRCC45 suggest that it may provide accessory interactions with nucleosome-linked ubiquitin chains that contribute to their efficient processing. Our data also suggest how ataxia telangiectasia mutated (ATM)-dependent BRCA1 dimerization may stabilize self-association of the entire BRCA1-A complex.

INTRODUCTION

Among the various types of genotoxic DNA lesions encountered by human cells, double-stranded DNA breaks (DSBs) are the most deleterious, because failure to faithfully repair them can result in a variety of mutations, including chromosomal rearrangements that are characteristic of cancer cells. The repair of DSBs is achieved by two mechanistically distinct processes: non-homologous end-joining (NHEJ) and homologous recombination (HR). NHEJ is a rapid error-prone repair mechanism whereby broken ends are directly ligated. In contrast, homologous recombination (HR) utilizes a sister chromatid to restore any lost or damaged information in an error-free process that can only be deployed in the S and G2 phases of the cell cycle when sister chromatids are present (Daley et al., 2014). A key player in HR repair is the tumor suppressor BRCA1 (breast cancer susceptibility protein 1), which, along with Bard1, is found in three distinct complexes (BRCA1-A, -B, and –C) that are distinguished by virtue of different phospho-dependent interactions of the Brca1 BRCT-repeats with pSPxF motifs in Abraxas (A), Bach1/FancJ (B), or CtIP (C) (Wang, 2012). Of these, BRCA1-A binds to chromatin regions flanking DSBs in a manner dependent upon a cascade of phosphorylation and ubiquitination events initiated by the MRN complex (Mre11-Rad50-NBS1). MRN senses the DSB and recruits and activates ATM (ataxia telangiectasia mutated), which, in turn, phosphorylates histone H2AX on Ser139 (Rogakou et al., 1998). Phosphorylated H2AX (γ-H2AX) is recognized by the BRCT-repeats of MDC1 (mediator of DNA damage checkpoint 1) (Lou et al., 2006; Stucki et al., 2005), which, itself, is a substrate of ATM. The subsequent MDC1-dependent recruitment of the ubiquitin E3 ligases RNF8 and RNF168 together with the E2-conjugating enzyme UBC13/MMS2 ultimately results in K63-linked polyubiquitination of histones H2A and H2AX (Al-Hakim et al., 2010; Kolas et al., 2007; Panier and Durocher, 2009). These ubiquitin (Ub) chains then provide the binding site for the ubiquitin-interacting motif (UIM) of the fifth BRCA1-A component, Rap80 (Sato et al., 2009). In addition to Rap80 and the BRCA1/BARD1 heterodimer, the BRCA1-A complex further comprises a catalytic deubiquitinase (DUB) core assembly of Abraxas/CCDC98, BRCC36, BRCC45/BRE, and Merit40/Nba1 (Feng et al., 2009; Hu et al., 2011; Shao et al., 2009b; Wang et al., 2009) that specifically targets and removes K63-linked ubiquitin chains from histones flanking the DSB, thus antagonizing the Mdc1/RNF8/RNF168 cascade and suppressing repair by HR (Shao et al., 2009a; Wang et al., 2007). BRCC36, BRCC45, and Merit40 proteins are also found in complex with an Abraxas paralog, Abro1/KIAA0157 (Feng et al., 2010; Patterson-Fortin et al., 2010), and an adaptor

This is an open access article under the CC BY license (http://creativecommons.org/licenses/by/4.0/).

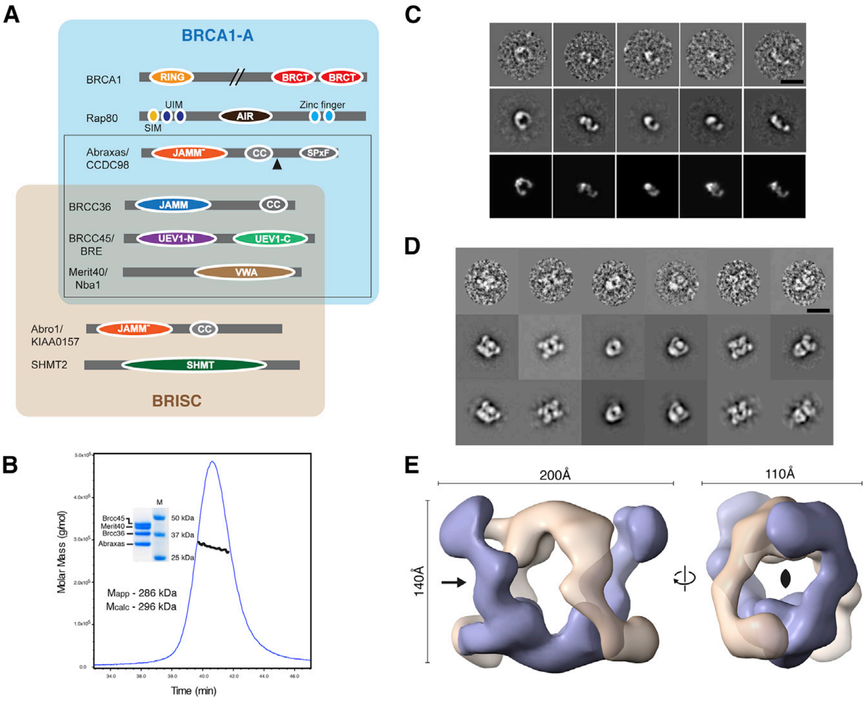

Figure 1. The BRCA1-A Complex

(A) Schematic representations of all known components of the BRCA1-A and BRISC complexes showing the location of known domains and relevant sites of post-translational modification. The Abraxas, BRCC36, BRCC45, and Merit40 components used for structural analysis are boxed, and the domain coloring is maintained throughout. The C-terminal truncation position of the Abraxas construct used for further analysis is shown by the black triangle. The asterisk denotes BRCC36 as the enzymatically active subunit.

(B) SEC-MALLS and SDS-PAGE analysis (inset) analysis of the purified BRCA1-A core complex. UV absorbance at 280 nm is shown in blue, and the weight-averaged molecular mass across the peak is shown in black.

(C) Gallery of individual particles from non-crosslinked BRCA1 complex (upper panel) contributing to a corresponding selection of 2D averages (middle panel). The number of particles contributing to each class sum is 65, 96, 123, 127, and 104 from left to right. Lower panel: alignment of reprojections of the segment-defined structure corresponding to half of the crosslinked BRCA1 complex.

(D) Gallery of individual particles from the crosslinked BRCA1 complex (upper) with a corresponding selection of 2D averages (middle) used to calculate an initial 3D structure and corresponding reprojections of this structure (lower). The number of particles contributing to each class sum is 378, 265, 278, 277, 224, and 151 from left to right.

(E) Surface representation of the three-dimensional map of the crosslinked BRCA1 complex with segmentation-defined half-complex maps (related by C2 symmetry) shown in gray and purple. The contour level for the surface encloses a volume corresponding to 580 kDa (assuming 1.21 Da/Å^3).

Scale bars, 20 nm (C and D).

protein, SHMT2, that is unrelated to Rap80 (Zheng et al., 2013) (Figure 1A). Together, these form a distinct cytoplasmic DUB assembly, BRISC (BRCC36 isopeptide complex), that plays a role in the deubiquitination and stabilization of the type I interferon receptor (Zheng et al., 2013). Within both BRCA1-A and BRISC, catalytic activity is provided by BRCC36, a member of the JAMM/MPN+ (Jacq et al., 2013; Komander et al., 2009a) Zn^{2+} metalloprotease-like class of deubiquitinases. Like many other members of this family, BRCC36 is activated though pairing with a partner protein containing an inactive MPN domain (MPN^{-}), Abraxas in BRCA1-A, and Abro1 in BRISC. Structural and biochemical analysis of insect Abro1/BRCC36 complexes has elegantly shown how BRCC36 is allosterically activated by Abro1 binding (Zeqiraj et al., 2015) and how this heterodimeric complex self-associates to form a tight tetrameric assembly, referred to as a "superdimer". Nonetheless, a molecular description of the overall architecture of either the BRCA1-A or BRISC complexes remains elusive. Here, we present the structure of a catalytically active BRCA1-A core complex consisting of Abraxas, Brcc36, Brcc45, and Merit40 determined by single particle negative-stain electron microscopy (EM). Our data provide important insights into the structural and functional contributions of constituent subunits to BRCA1-A function as a histone deubiquitinase and further suggest a structural basis for ATM-dependent BRCA1 dimerization (Wu et al., 2016) in regulation of the BRCA1-A complex after DNA-damage.

RESULTS

In order to produce BRCA1-A complex samples for structural and biochemical studies, we took a variety of approaches focused on co-expression of all or combinations of each of the subunits and appropriate deletion variants in both *Escherichia coli* and insect cell systems. These experiments were, in turn, guided by the known domain organization of the component proteins and available information about their possible arrangements within the holo-complex (Figure 1A). We were successful in reconstituting a four-component "core" assembly of Abraxas/BRCC36/BRCC45/MERIT40 from two bacterially expressed subcomplexes containing BRCC36 and an Abraxas fragment (residues 1–269), and full-length BRCC45/MERIT40. BRCC45/MERIT40 formed a soluble and highly stable association with 1:1 stoichiometry as judged from analysis by multi-angle laser light scattering with size-exclusion chromatography (SEC-MALLS) (Figure S1A). However, and in contrast to insect Abro1/BRCC36 complexes described previously (Zeqiraj et al., 2015), the human Abraxas/BRCC36 subcomplex behaved as a soluble aggregate that could not be further purified, but was, nonetheless, able to form a stable monodispersed and stoichiometric assembly when purified in combination with BRCC45/MERIT40. Thus, it seems likely that the apparent requirement of BRCC45 for DUB activity of the BRCA1-A complex, but not BRISC (Patterson-Fortin et al., 2010), is not due to major structural differences between the two complexes, but merely reflects

a specific stabilizing effect of BRCC45 on Abraxas/BRCC36 complexes that is not required by Abro1/BRCC36. Regardless, MALLS analysis of the reconstituted four-component BRCA1-A complex reported an apparent molecular mass of 280 kDa (Figure 1B), suggestive of a dimer of Abraxas/BRCC36/BRCC45/MERIT40 heterotetramers and consonant with the "super-dimer" originally described for insect Abro1/BRCC36 heterodimers (Zeqiraj et al., 2015). Furthermore, this purified "super-tetrameric" complex (the "2×4" complex) displayed substantial deubiquitinase activity on K63-linked ubiquitin substrates (Figure S1B), consistent with previous observations that Rap80 is not required for enzymatic activity (Patterson-Fortin et al., 2010).

Initial analysis by negative-stain electron microscopy showed substantial sample heterogeneity, consistent with our difficulties in producing well-diffracting crystals. Nonetheless, they did reveal the presence of particles with a striking horseshoe or V-shaped appearance from which we could generate coherent averages (Figure 1C, top and center panels). In order to stabilize the complex and improve the quality of the particle fields, we used the Grafix cross-linking procedure (Stark, 2010). This resulted in a much-improved and essentially homogeneous field of particles that were, somewhat surprisingly, larger and more globular than those seen in the non-crosslinked preparations (Figures 1D and S1C). The resulting reconstruction produced a volume apparently formed from two interwoven V-shaped assemblies closely resembling those seen in the initial specimens (Figure 1E). This was confirmed by extracting a single V-shaped sub-volume and comparing suitably aligned reprojections with the initial class sums generated from the un-crosslinked samples (Figure 1C, bottom panel). Overall, the particle (the "4×4" complex) displays clear C2 symmetry and pseudo-D2 symmetry that is broken by a markedly different arrangement of each of the stalk regions with respect to the base of the V-shaped sub-volumes.

In constructing a molecular model of the "2×4" complex, we first docked the X-ray structure of the Abro1/BRCC36 super-dimer (PDB: 5CW3) into the base of the trapezoid such that the local 2-fold symmetry axis was coincident with that of the EM volume (Figure 2A). This produced an excellent fit, leaving a large unfilled volume protruding from each side of the base. As mentioned, Abraxas and Abro1 each constitute the major scaffolding components of the nuclear and cytoplasmic versions of these DUB complexes, respectively. Their cognate Rap80 and SHMT2 adaptor proteins do not appear to share the same binding sites and appear to be specific to their respective core assemblies (Zheng et al., 2013). However, since both Abraxas and Abro1 each bind to both BRCC36 and BRCC45 in their respective DUB complexes, we surmised that the interaction surfaces for these components should show the highest degree of conservation between the two paralogs. This, indeed, proved to be the case and beyond the known Brcc36 binding surface, the only other region of significant homology, covers a surface on Abraxas that almost exactly coincides with the stalks of the EM reconstruction, therefore defining the likely interacting region for BRCC45 (Figure 2B). Furthermore, the juxtaposition of the arm region with Abraxas is in agreement with the experimental determination of the absolute hand of the reconstruction by tilt-series analysis (Figure S2A). By way of confirmation, we carried out native nanospray mass spectrometry on a subcomplex consisting of only the Abraxas, BRCC45, and MERIT40 components that we were able to purify, albeit in limited quantities (Figure 2C). In addition to the heterotrimeric species, partial dissociation was observed, enabling a pairwise interaction map to be generated. These data clearly show that incorporation of BRCC45 and MERIT40 into the BRCA1-A complex is independent of BRCC36, as reported previously (Ng et al., 2016), and are consistent with a linear association, whereby BRCC45 forms a bridge between MERIT40 and the Abraxas scaffold. The VWA (von Willebrand factor type A) domain core of MERIT40 was therefore modeled into the terminal regions of the arm segments using the crystal structure of the homologous domain from Rpn10 (PDB: 2X5N; Figure S3A). This places MERIT40 distant from the catalytic sites, in agreement with the observations that its absence does not significantly affect DUB activity in vitro (Patterson-Fortin et al., 2010). Finally, the N- and C-terminal UEV (Ubc13-E2 variant) domains were modeled using the MMS2 UEV domain coordinates (PDB: 1J7D; Figure S3B) into the stalk regions contacting Abraxas and Merit40, respectively (Figure 2D), again in accord with previous deletion studies (Hu et al., 2011). Only two significant regions of residual density remain. The first is located around the MERIT40 VWA domain, presumably arising from the N- and C-terminal segments not present in the coordinates used for modeling. The second forms the "elbow" region between the N- and C-terminal UEV domains of each BRCC45 component, which we assume delineates the position of the intervening linker.

The location of the BRCC45 UEV domains in the structure is intriguing. UEV domains are most often found in E2 ubiquitin-conjugating complexes, where they can act catalytically in the transthioesterification reaction or provide a non-catalytic ubiquitin (Ub)-binding function that may help to determine linkage specificity (Komander et al., 2009a). Indeed, using the purified BRCC45/MERIT40 complex, we have been able to determine an affinity for binding to K63-linked di-ubiquitin (di-Ub) of around 17 μM using biolayer interferometry (Figure 3A), consistent with previous glutathione S-transferase (GST) pull-down experiments (Wang et al., 2009). No VWA domains have been reported to have Ub-binding activity. Therefore, it seems likely that one or both BRCC45 UEVs play an accessory role in substrate tethering by the BRCA1-A core. With this in mind, structural superposition of one of the BRCC36 molecules from the docked superdimer with a K63-Ub_2 complex of the related MPN^+ DUB, AMSH-LP (PDB: 2ZNV), enables us to position the "proximal" and "distal" Ub molecules with respect to the BRCC36 active site (Figure 3B; Zeqiraj et al., 2015). In this context, the proximal Ub is closest to the substrate and supplies Lys63, which is linked to the C terminus of the distal Ub through the scissile isopeptide bond. In this arrangement, it is clear that additional Ub units on the distal end of the chain are directed out into bulk solvent (Figure 3C). However, the position of the proximal Ub nestles against the surface of BRCC36, close to the interface with Abraxas. Extending the chain on both sides using the structure of a K63-linked-Ub dimer (PDB: 2JF5) shows that the UEV domains from BRCC45 are in position to provide a potentially extensive accessory interaction

OPEN ACCESS
CellPress

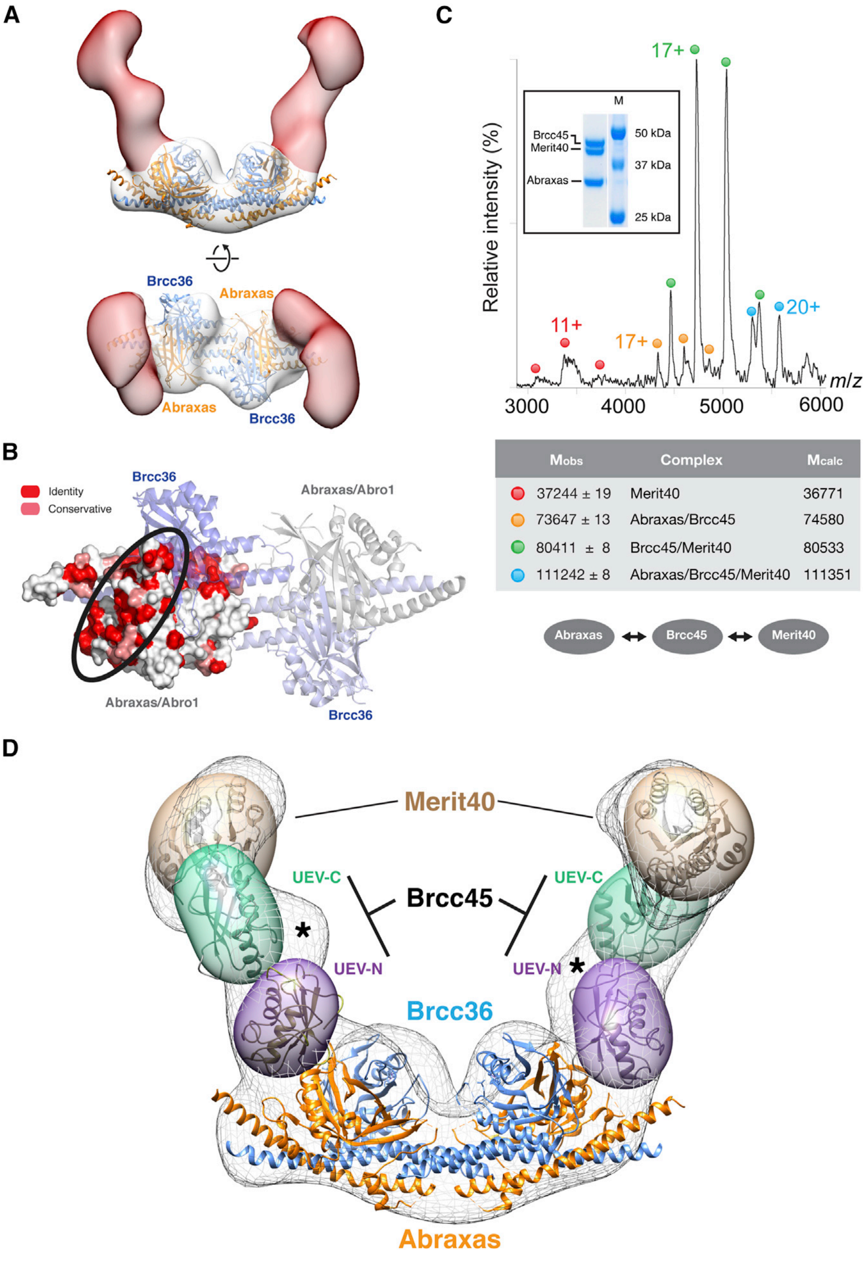

Figure 2. A Model for the BRCA1-A Core Complex

(A) Orthogonal views of the 2×4 complex subvolume. Docking of the coordinates of the Abro1/BRCC36 superdimer (Zeqiraj et al., 2015) into the base (white surface) defines the two-arm segments (red surface) as the location of the BRCC45 and MERIT40 components. The bottom image shows the broken symmetry at the ends of the complex.

(B) Mapping of sequence alignment of human Abraxas and Abro1 onto the Abro1/BRCC36 structure reveals a conserved surface patch that defines the likely site of BRCC45 binding.

(C) Native nanospray MS spectrum of the trimeric Abraxas/BRCC45/MERIT40 complex (inset). Experimental and calculated molecular masses for each of the observed species are shown in the middle panel, revealing that BRCC45 forms a bridge between Abraxas and MERIT40 subunits.

(D) Model of the BRCA1-A 2×4 complex showing the positions of the Abraxas and BRCC36 superdimer in the base, the approximate locations of the BRCC45 UEV domains in the arm regions, and the VWA domain of MERIT40 at the apices. BRCC45 and MERIT40 components are shown as semi-transparent ellipsoidal volumes with ribbon representations of the coordinates used for modeling included for size comparison only. The asterisk shows the position of the elbow region, which, presumably, contains the linking region between the UEV domains of BRCC45. The docking of atomic coordinates was performed with UCSF Chimera (Pettersen et al., 2004).

surface for ubiquitin molecules on the proximal, but not the distal, side of the cleavage site.

DISCUSSION

Here, we have described a structural and biochemical analysis of a core assembly from the human BRCA1-A deubiquitinase complex. Single-particle negative-stain EM reconstructions reveal the organization of the four components, Abraxas, BRCC36, BRCC45, and MERIT40, within an unusual V-shaped architecture. The structure now provides a molecular framework within which functional observations for both the nuclear (BRCA1-A) and cytoplasmic (BRISC) DUB complexes can be interpreted and insights into how these complex deubiquitinase assemblies may be regulated.

Previous structural studies have clearly shown how the BRISC subunit Abro1 binds to and allosterically activates the DUB activity of BRCC36 and how this heterodimer further self-associates to form a "superdimeric" assembly (Zeqiraj et al., 2015). Given the high degree of sequence conservation between Abro1 and Abraxas, the similarities in subunit composition, and, presumably, overall structures of BRCA1-A and BRISC, it seems likely that the remaining common components, BRCC45 and MERIT40, perform closely related functions in both contexts that, nonetheless, remain poorly defined at a molecular level. In the case of BRCC45, our structural analysis now suggests that at least one of those common functionalities may be in Ub chain interactions. Modeling of the path of the bound Ub substrate (Figure 3B) shows that, beyond the Ub pair bound at the BRCC36 active site, additional interactions with the core assembly are likely to occur between the UEV domains of BRCC45 and ubiquitin units on the side of the chain that is ultimately linked to the modified nucleosome. Unlike K48-linked Ub, which shows substantial and conformationally restrictive Ub-Ub interactions, K63-linked Ub

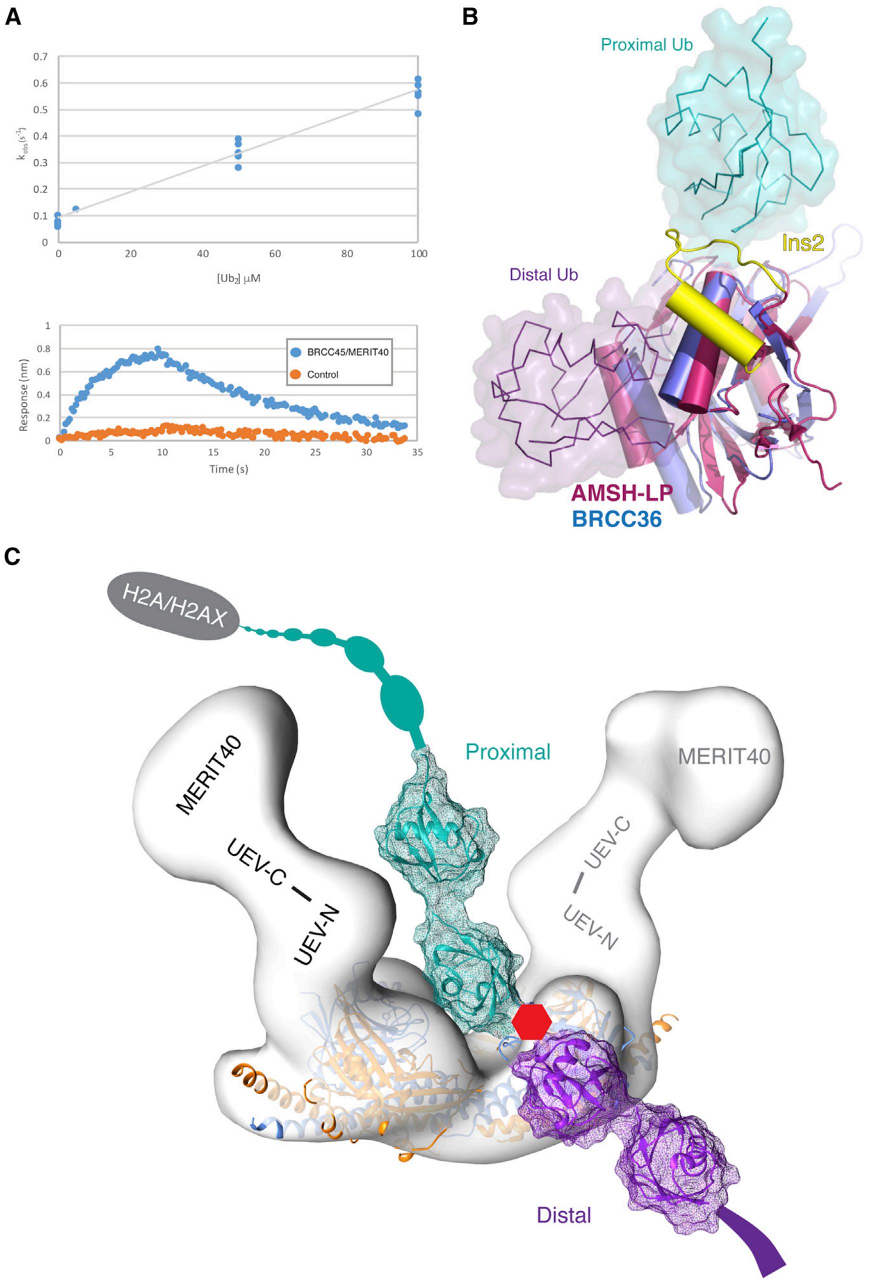

Figure 3. Ubiquitin Binding by BRCA1-A

(A) Upper panel: binding of di-ubiquitin to the BRCC45/MERIT40 subcomplex measured by biolayer interferometry. The top panel shows the pseudo first-order association constant (K_{obs}) plotted against K63-linked Ub_2 concentration. The dissociation equilibrium constant (K_d) of 17 uM was determined from the ratio of k_{off} and k_{on}. Lower panel: association and dissociation phases in the raw kinetic trace for Ub_2 binding to a probe linked to the BRCC45/MERIT40 complex (blue) and a non-derivatized probe (orange).

(B) Superposition of the AMSH-LP/K63-Ub_2 complex with the docked Abro1/BRCC36 structure shows the approximate positions of the proximal (teal) and distal (purple) Ub molecules. As previously noted (Zeqiraj et al., 2015), the Ins-2 loop that packs against the proximal Ub in AMSH-LP (yellow) is absent in BRCC36.

(C) The alignment in (A) and the structure of K63-linked di-ubiquitin were used to model an extended Ub chain on the proximal and distal sides of the cleavage site into the 2×4 complex (white surface). Abraxas and BRCC36 are shown in gold and blue, respectively.

chains are considerably more flexible (Komander et al., 2009b; Weeks et al., 2009). While Ub interactions with either of the two symmetrically arranged BRCC36 components in the 2×4 assembly is feasible, closer inspection of our modeled Ub complex (Figure 3B) suggests the intriguing possibility that BRCC45 tethers the ubiquitin chain substrate being processed by the opposing BRCC36 subunit in the 2×4 complex. This is entirely consistent with the somewhat unexpected "half-of-sites" activity reported previously for superdimeric Abro1/BRCC36 and goes some way to rationalizing the overall architecture of the octameric 2×4 complex, with each half of the complex contributing different but complementary functions. In addition, the utilization of accessory interactions with Ub units on the proximal, but not distal, side may act to retain the DUB complex close to its substrate following isopeptide bond cleavage, potentially facilitating subsequent processing events.

In contrast to BRCC45, the role of MERIT40 is less clear. Our structural data place it at the ends of the two arms of the V-shaped complex, in contact with BRCC45, but remote from the BRCC36, active sites. The flexible association inferred from the EM class sums derived from the un-crosslinked 2×4 complex is consistent with MERIT40 tethering to BRCC45 through a PxxR motif in a C-terminal extension, rather than the VWA domain itself (Hu et al., 2011; Shao et al., 2009b). Loss of MERIT40 appears to destabilize BRCC45 and the BRCA1-A complex, in general, through an unknown mechanism (Feng et al., 2009; Shao et al., 2009b; Wang et al., 2009). However, the apparent requirement of MERIT40 for correct localization of BRCA1-A to DNA-damage foci may reflect a more direct contribution through interactions of its VWA domain or N-terminal region with, as yet, unidentified partners. Indeed, a model of the full hexadecameric 4×4 complex (Figure 4A) suggests that MERIT40 mediates much of the overall apparent interaction surface between the constituent 2×4 assemblies by stacking against BRCC36 from the opposing complex (Figure 4A). Chemical cross-linking can often identify functionally significant complexes formed between molecules that otherwise interact weakly or transiently, raising the

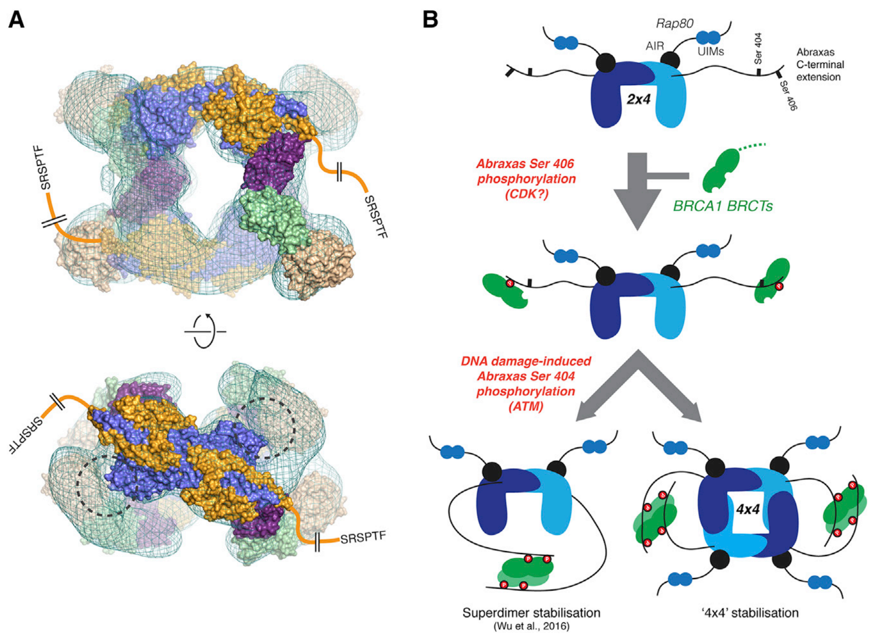

Figure 4. Architecture of the 4×4 Complex and Brca1 Dimerization

(A) Orthogonal views of a model of the complete crosslinked 4×4 particle generated from two copies of the 2×4 complex (colored as in Figure 1A) docked into the EM reconstruction (cyan mesh). Positions of visible C-termini of the Abraxas subunits are indicated. Points of closest approach between BRCC36 (blue) and MERIT40 (wheat are highlighted with dashed circles) are shown.

(B) Schematic showing a potential cellular role for ATM-dependent dimerization of BRCA1 in interactions with the 2×4 (lower left) and the 4×4 (lower right) complexes. (UIM, ubiquitin-interacting motif; AIR, Abraxas-interacting region.)

interesting possibility that a 4×4-like BRCA1-A supercomplex may have a physiological role. In this regard, it has been shown recently that BRCA1 recruitment is stabilized by a DNA damage-dependent head-to-tail dimerization of its BRCT-repeat domain mediated by dual phosphorylation of the Abraxas C terminus (Wu et al., 2016; Figure 4B). The location of the Abraxas C-termini in the 4×4 particle now suggests that a similar set of phospho-dependent interactions might stabilize this higher-order arrangement (Figure 4B), perhaps providing an opportunity for indirect BRCA1-dependent regulation of deubiquitinase activity. Furthermore, and given that the 4×4 complex would also contain four Rap80 molecules, BRCA1-mediated self-association might also be expected to increase overall avidity for ubiquitinylated chromatin, potentially allowing fine-tuning of the amplitude and duration of BRCA1-A activity during the repair process.

EXPERIMENTAL PROCEDURES

Expression and Purification

The BRCA1-A complex core was expressed as two two-component complexes in the *E. coli* strain BL21(DE3): Abraxas (1-269, N-terminal GST-tag) with BRCC36 (FL, C-terminal His tag) and BRCC45 (4-383, N-terminal Streptavidin tag) and Merit40 (FL, N-terminal His tag), respectively. Pelleted cells of both complexes were resuspended together in buffer I (40 mM Tris pH 8.0, 100 mM NaCl, 1 mM TCEP) and lysed using a cell disruptor. Cleared lysate was loaded on to glutathione-Sepharose 4 Fast Flow (GE Healthcare) equilibrated with buffer I. Beads were washed with 40 mM Tris pH 8.0, 1 M NaCl, 1 mM TCEP, and the complex was eluted with buffer I supplemented with 20 mM reduced L-glutathione. Pooled fractions were dialysed against buffer I. The tags of Abraxas, BRCC45, and Merit40 were cleaved with GST-tagged 3C protease, and the complex was repurified on GST Sepharose to remove the protease and any uncleaved complex. Following final purification by MonoQ anion exchange chromatography and size exclusion chromatography on Superose 6, the BRCA1-A core complex was concentrated to 20 mg/mL, shock frozen in liquid nitrogen, and stored at −80°C.

Cross-Linking of the BRCA1-A Complex Core for Negative-Stain EM

For the cross-linking of the BRCA1-A complex core, the GraFix method was used (Stark, 2010). Cross-linking was carried out in buffer IV (40 mM HEPES, pH = 7.5, 100 mM NaCl, 1 mM TCEP). The 4-component complex (~3 mg/mL) was loaded onto a 10%–30% sucrose/0%–0.1% glutaraldehyde gradient and centrifuged in a SW55 rotor for 16 hr at 30,000 rpm at 4°C using a Beckman Coulter Optima L90K ultracentrifuge. Gradient fractions containing the crosslinked complex were identified by SDS-PAGE and pooled. Remaining glutaraldehyde was inactivated by the addition of Tris pH 7.5 to a final concentration of 10 mM, and the combined inactivated fractions were "polished" by Superose 6 10/300 chromatography (GE Healthcare).

Sample Preparation for Negative-Stain EM

2 μL of protein (20 μg/mL) was applied to the clean side of carbon on mica (carbon/mica interface), and the carbon layer was then floated onto PBS and recovered with a 400 mesh copper grid (TAAB). The grid was immediately floated onto 1% phosphotungstate solution (pH 7.4) for staining.

Data Collection: Electron Microscopy

Electron microscopy was performed on an FEI Spirit TWIN transmission electron microscope using a tungsten filament source and operating at 120 kV. Images were recorded on an Eagle 2K camera at 52 K magnification, corresponding to a pixel size of 4.3 Å/pixel at an electron dose of less than 30 $e^-/Å^2$ and with a defocus of −1.5 μm.

Image Analysis and 3D Reconstruction

Particles were semi-automatically extracted in 120×120 pixel boxes using the EMAN2 boxer swarm tool (Tang et al., 2007) and followed by manual examination. The non-crosslinked dataset consisted of 1,592 particles, and the crosslinked dataset consisted of 8,393 particles. Images were filtered to the first zero of the contrast transfer function (22 Å). Reference-free 2D alignment, classification, and averaging of particle images were carried out using IMAGIC software (van Heel et al., 1996). Initial maps of the crosslinked complex assuming C2 symmetry were calculated using angular reconstitution and improved by anchor-set refinement. Deviations from an apparent D2 arrangement for the reconstructed volume were significant and application of higher symmetry was not justified. This map was masked and filtered to 45 Å and then subsequently refined using Frealign (v9.11) (Lyumkis et al., 2013), increasing the high-resolution limit of the data used for refinement in 5 Å steps to 30 Å. To validate the Fourier shell correlation (FSC) curve, the final round of refinement was repeated using image data with phases greater than 30 Å randomized (Chen et al., 2013). The FSC between two maps calculated from different subsets of particle images provided a resolution estimate of 24.8 Å

using the 0.143 threshold criterion (Rosenthal and Henderson, 2003). A final refinement to 27 Å produced no further increase in resolution (Figure S2B). Maps were displayed and the docking of atomic coordinates was performed with UCSF Chimera (Pettersen et al., 2004). Segmented maps produced with the Segger-option were used for estimating the volume of the half map to approximate the non-crosslinked complex. Volumes for the UEV and VWA domains, blurred to 60 Å, were generated using pdb2mrc in EMAN.

SEC MALLS Analysis

The BRCA1-A complex core and the two-component complex BRCC45ΔN4/Merit40 were analyzed by SEC coupled to multiangle laser light scattering (SEC-MALLS). Samples (100 μL) were run at concentrations of 1.2 mg/mL and 1.0 mg/mL, respectively, on a Superose 6 10/300 GL column mounted on a Jasco HPLC equilibrated in 40 mM Tris pH 8.0, 100 mM NaCl, and 1 mM TCEP at a flow rate of 0.5 mL/min. The scattered light intensity and protein concentration of the column eluate were recorded using a DAWN HELEOS laser photometer and an OPTILAB-rEX differential refractometer (ΔRI) (dn/dc = 0.186), respectively. Data were analyzed using ASTRA software version 6.0.3 (Wyatt Technology).

Native Mass Spectroscopy

Samples were diluted to a concentration of 3 μM protein in 150 mM ammonium acetate pH 7.6 and further buffer-exchanged into 150 mM ammonium acetate using a Bio-Spin 6 (Bio-Rad) column. The desalted samples were loaded into in-house-prepared gold-coated glass capillaries. Nano-electrospray mass spectrometric analyses were performed under native conditions on a hybrid quadrupole time-of-flight mass spectrometer previously modified for high mass transmission. Typical conditions for analysis used capillary, cone, and CID voltages of 1.6 kV, 80 V, and 20 V. Backing pressure was set to 4.08e-3 bar.

Binding Analysis

K63-linked di-ubiquitin binding to BRCC45/Merit40 was measured using an Octet RED biolayer interferometer (Pall ForteBio). His-tagged BRCC45/Merit40 was immobilized on anti-penta His biosensors (Pall ForteBio) at a concentration of approximately 25 μg/mL. The binding of K63-linked di-ubiquitin (at 5–100 μM) to immobilized BRCC45/MERIT40 was measured at 25°C with a 120 s association step, followed by a 200 s dissociation step in a buffer containing 40 mM Tris pH 8.0, 100 mM NaCl, and 1 mM TCEP. The association phase was analyzed as a single exponential function using in-house software, and a plot of the observed rate (k_{obs}) versus ligand concentration gave the association and dissociation rate constants (k_{on} and k_{off}) as the slope and intercept, respectively. The equilibrium dissociation constant was determined as k_{off}/k_{on}.

ACCESSION NUMBERS

The accession number for the density map for the BRCA1-A core complex reported in this paper is EM Data Bank: EMD-3463.

SUPPLEMENTAL INFORMATION

Supplemental Information includes Supplemental Experimental Procedures and three figures and can be found with this article online at http://dx.doi.org/10.1016/j.celrep.2016.11.063.

AUTHOR CONTRIBUTIONS

O.J.P.K. designed and produced expression constructs; performed protein expression and purification, mutagenesis, and biochemical assays; and contributed to model-building and manuscript preparation. P.B.M. carried out analysis of EM data and structure determination and contributed to manuscript preparation. L.J.C. performed electron microscopy. S.R.W. established the GraFix procedure and assisted with complex preparation. S.R.M. carried out Octet binding experiments. N.A.P. carried out native mass spectrometry measurements. J.L. assisted with bacterial and insect cell expression experiments. C.V.R., P.B.R., and S.J.S. analyzed the data. S.J.S. conceived the project, contributed to model-building, made the figures, and wrote the manuscript.

ACKNOWLEDGMENTS

The authors wish to thank Vangelis Christodoulou for the initial test expressions of the BRCA1-A complex components, Ian Taylor for help with MALS experiments, and Shahid Mehmood for assistance with the measurement and analysis of native mass spectroscopy data. Work in the C.V.R. laboratory was funded by a Wellcome Trust Investigator award (104633/Z/14/Z). S.J.S. and P.B.R. are supported by the Francis Crick Institute, which receives its core funding from Cancer Research UK (FC001156 and FC001143), the UK Medical Research Council (FC001156 and FC001143), and the Wellcome Trust (FC001156 and FC001143).

Received: October 11, 2016
Revised: November 10, 2016
Accepted: November 21, 2016
Published: December 20, 2016

REFERENCES

Al-Hakim, A., Escribano-Diaz, C., Landry, M.-C., O'Donnell, L., Panier, S., Szilard, R.K., and Durocher, D. (2010). The ubiquitous role of ubiquitin in the DNA damage response. DNA Repair (Amst.) *9*, 1229–1240.

Chen, S., McMullan, G., Faruqi, A.R., Murshudov, G.N., Short, J.M., Scheres, S.H.W., and Henderson, R. (2013). High-resolution noise substitution to measure overfitting and validate resolution in 3D structure determination by single particle electron cryomicroscopy. Ultramicroscopy *135*, 24–35.

Daley, J.M., Gaines, W.A., Kwon, Y., and Sung, P. (2014). Regulation of DNA pairing in homologous recombination. Cold Spring Harb. Perspect. Biol. *6*, a017954–a017954.

Feng, L., Huang, J., and Chen, J. (2009). MERIT40 facilitates BRCA1 localization and DNA damage repair. Genes Dev. *23*, 719–728.

Feng, L., Wang, J., and Chen, J. (2010). The Lys63-specific deubiquitinating enzyme BRCC36 is regulated by two scaffold proteins localizing in different subcellular compartments. J. Biol. Chem. *285*, 30982–30988.

Hu, X., Kim, J.A., Castillo, A., Huang, M., Liu, J., and Wang, B. (2011). NBA1/MERIT40 and BRE interaction is required for the integrity of two distinct deubiquitinating enzyme BRCC36-containing complexes. J. Biol. Chem. *286*, 11734–11745.

Jacq, X., Kemp, M., Martin, N.M.B., and Jackson, S.P. (2013). Deubiquitylating enzymes and DNA damage response pathways. Cell Biochem. Biophys. *67*, 25–43.

Kolas, N.K., Chapman, J.R., Nakada, S., Ylanko, J., Chahwan, R., Sweeney, F.D., Panier, S., Mendez, M., Wildenhain, J., Thomson, T.M., et al. (2007). Orchestration of the DNA-damage response by the RNF8 ubiquitin ligase. Science *318*, 1637–1640.

Komander, D., Clague, M.J., and Urbé, S. (2009a). Breaking the chains: structure and function of the deubiquitinases. Nat. Rev. Mol. Cell Biol. *10*, 550–563.

Komander, D., Reyes-Turcu, F., Licchesi, J.D.F., Odenwaelder, P., Wilkinson, K.D., and Barford, D. (2009b). Molecular discrimination of structurally equivalent Lys 63-linked and linear polyubiquitin chains. EMBO Rep. *10*, 466–473.

Lou, Z., Minter-Dykhouse, K., Franco, S., Gostissa, M., Rivera, M.A., Celeste, A., Manis, J.P., van Deursen, J., Nussenzweig, A., Paull, T.T., et al. (2006). MDC1 maintains genomic stability by participating in the amplification of ATM-dependent DNA damage signals. Mol. Cell *21*, 187–200.

Lyumkis, D., Brilot, A.F., Theobald, D.L., and Grigorieff, N. (2013). Likelihood-based classification of cryo-EM images using FREALIGN. J. Struct. Biol. *183*, 377–388.

Ng, H.-M., Wei, L., Lan, L., and Huen, M.S.Y. (2016). The Lys63-deubiquitylating Enzyme BRCC36 Limits DNA Break Processing and Repair. J. Biol. Chem. *291*, 16197–16207.

Panier, S., and Durocher, D. (2009). Regulatory ubiquitylation in response to DNA double-strand breaks. DNA Repair (Amst.) *8*, 436–443.

Patterson-Fortin, J., Shao, G., Bretscher, H., Messick, T.E., and Greenberg, R.A. (2010). Differential regulation of JAMM domain deubiquitinating enzyme activity within the RAP80 complex. J. Biol. Chem. *285*, 30971–30981.

Pettersen, E.F., Goddard, T.D., Huang, C.C., Couch, G.S., Greenblatt, D.M., Meng, E.C., and Ferrin, T.E. (2004). UCSF Chimera–a visualization system for exploratory research and analysis. J. Comput. Chem. *25*, 1605–1612.

Rogakou, E.P., Pilch, D.R., Orr, A.H., Ivanova, V.S., and Bonner, W.M. (1998). DNA double-stranded breaks induce histone H2AX phosphorylation on serine 139. J. Biol. Chem. *273*, 5858–5868.

Rosenthal, P.B., and Henderson, R. (2003). Optimal determination of particle orientation, absolute hand, and contrast loss in single-particle electron cryomicroscopy. J. Mol. Biol. *333*, 721–745.

Sato, Y., Yoshikawa, A., Mimura, H., Yamashita, M., Yamagata, A., and Fukai, S. (2009). Structural basis for specific recognition of Lys 63-linked polyubiquitin chains by tandem UIMs of RAP80. EMBO J. *28*, 2461–2468.

Shao, G., Lilli, D.R., Patterson-Fortin, J., Coleman, K.A., Morrissey, D.E., and Greenberg, R.A. (2009a). The Rap80-BRCC36 de-ubiquitinating enzyme complex antagonizes RNF8-Ubc13-dependent ubiquitination events at DNA double strand breaks. Proc. Natl. Acad. Sci. USA *106*, 3166–3171.

Shao, G., Patterson-Fortin, J., Messick, T.E., Feng, D., Shanbhag, N., Wang, Y., and Greenberg, R.A. (2009b). MERIT40 controls BRCA1-Rap80 complex integrity and recruitment to DNA double-strand breaks. Genes Dev. *23*, 740–754.

Stark, H. (2010). GraFix: stabilization of fragile macromolecular complexes for single particle cryo-EM. Methods Enzymol. *481*, 109–126.

Stucki, M., Clapperton, J.A., Mohammad, D., Yaffe, M.B., Smerdon, S.J., and Jackson, S.P. (2005). MDC1 directly binds phosphorylated histone H2AX to regulate cellular responses to DNA double-strand breaks. Cell *123*, 1213–1226.

Tang, G., Peng, L., Baldwin, P.R., Mann, D.S., Jiang, W., Rees, I., and Ludtke, S.J. (2007). EMAN2: an extensible image processing suite for electron microscopy. J. Struct. Biol. *157*, 38–46.

van Heel, M., Harauz, G., Orlova, E.V., Schmidt, R., and Schatz, M. (1996). A new generation of the IMAGIC image processing system. J. Struct. Biol. *116*, 17–24.

Wang, B. (2012). BRCA1 tumor suppressor network: focusing on its tail. Cell Biosci. *2*, 6.

Wang, B., Matsuoka, S., Ballif, B.A., Zhang, D., Smogorzewska, A., Gygi, S.P., and Elledge, S.J. (2007). Abraxas and RAP80 form a BRCA1 protein complex required for the DNA damage response. Science *316*, 1194–1198.

Wang, B., Hurov, K., Hofmann, K., and Elledge, S.J. (2009). NBA1, a new player in the Brca1 A complex, is required for DNA damage resistance and checkpoint control. Genes Dev. *23*, 729–739.

Weeks, S.D., Grasty, K.C., Hernandez-Cuebas, L., and Loll, P.J. (2009). Crystal structures of Lys-63-linked tri- and di-ubiquitin reveal a highly extended chain architecture. Proteins *77*, 753–759.

Wu, Q., Paul, A., Su, D., Mehmood, S., Foo, T.K., Ochi, T., Bunting, E.L., Xia, B., Robinson, C.V., Wang, B., and Blundell, T.L. (2016). Structure of BRCA1-BRCT/Abraxas Complex Reveals Phosphorylation-Dependent BRCT Dimerization at DNA Damage Sites. Mol. Cell *61*, 434–448.

Zeqiraj, E., Tian, L., Piggott, C.A., Pillon, M.C., Duffy, N.M., Ceccarelli, D.F., Keszei, A.F.A., Lorenzen, K., Kurinov, I., Orlicky, S., et al. (2015). Higher-Order Assembly of BRCC36-KIAA0157 Is Required for DUB Activity and Biological Function. Mol. Cell *59*, 970–983.

Zheng, H., Gupta, V., Patterson-Fortin, J., Bhattacharya, S., Katlinski, K., Wu, J., Varghese, B., Carbone, C.J., Aressy, B., Fuchs, S.Y., and Greenberg, R.A. (2013). A BRISC-SHMT complex deubiquitinates IFNAR1 and regulates interferon responses. Cell Rep. *5*, 180–193.

Cell Reports, Volume *17*

Supplemental Information

Three-Dimensional Architecture of the Human BRCA1-A Histone Deubiquitinase Core Complex

Otto J.P. Kyrieleis, Pauline B. McIntosh, Sarah R. Webb, Lesley J. Calder, Janette Lloyd, Nisha A. Patel, Stephen R. Martin, Carol V. Robinson, Peter B. Rosenthal, and Stephen J. Smerdon

Supplemental Information

Three-dimensional architecture of the human BRCA1-A histone deubiquitinase core complex

Otto J.P. Kyrieleis, Pauline B. McIntosh, Sarah R. Webb, Lesley J. Calder, Janette Lloyd, Nisha A. Patel, Stephen R. Martin, Carol V. Robinson, Peter B. Rosenthal and Stephen J. Smerdon

Supplementary Experimental Procedures

Supplementary Figures S1, S2 & S3

Supplementary References

Supplementary Experimental Procedures

DUB activity assay

The DUB activity assay was carried out in 40 mM Tris pH=8.0, 100 mM NaCl, 1 mM TCEP. The active BRCA1 A complex core was combined with K63-linked penta-ubiquitin in a total volume of 15 µl and incubated at 37°C for 5, 10, 20, 30, 40, 50, 60, 90 and 120 min. The reaction was quenched by adding SDS sample buffer and by heating the sample to 100°C for 5 min. Samples were analysed by SDS-PAGE. The following enzyme concentrations were used in this DUB assay: [UB_5, K63-linked] =23.5 µM and [BRCA1-A] = 16.8 nM.

Tilt-pair analysis and determination of absolute hand

Tilt-pair images, at 20° and -20°, were obtained for the cross linked structure using the same grids and conditions as described above. Particle pairs were picked from tilted images using Xmipp tilt-pair picking function. Euler angles were assigned to both tilted and untilted particle stacks using 1 cycle of Frealign in mode 3 (global search) with the final map as a reference. Tilt-pair analysis (Rosenthal & Henderson, 2003) was performed using the tilt-service (Wasilewski and Rosenthal, 2014). The minimum in the tilt-pair parameter plot agreed with that expected for the known tilt-axis and tilt-angle used in the experiment confirmed by the presence of the expected defocus gradient in the image fields. However, the minimum was at a smaller angle than used in the experiment due to flattening of the particles in stain.

Figure S1

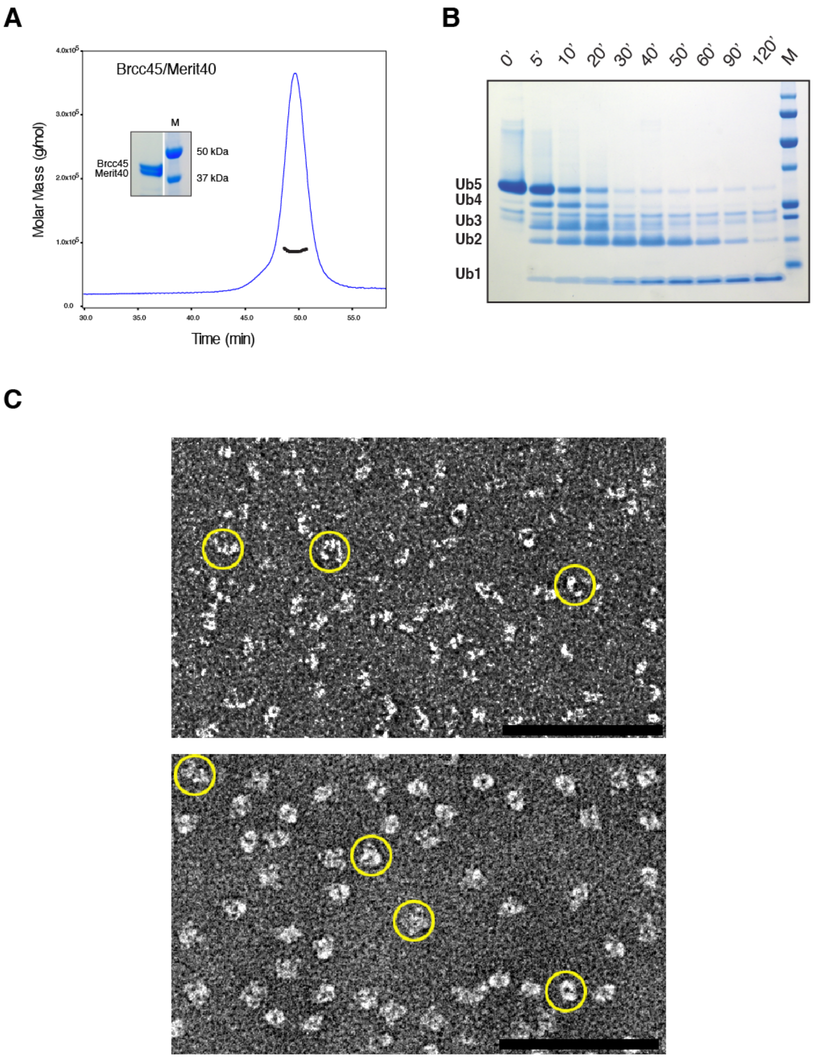

Figure S1 (Related to Figure 1) **BRCA1-A complex (A)** SEC-MALLS and SDS-PAGE analysis (inset) analysis of the purified BRCC45/MERIT40 complex. UV absorbance at 280 nm is shown in blue and the weight-averaged molecular mass across the peak in black. **(B)** Deubiquitinase assay of the BRCA1-A core complex with K63-linked penta-ubiquitin substrate analysed by SDS-PAGE. **(C)** Representative fields of raw negatively stained electron micrographs obtained from both un-crosslinked (Top) and crosslinked (Bottom) BRCA1-A complex, scale bars 100 nm. Representative views are circled.

Figure S2

A

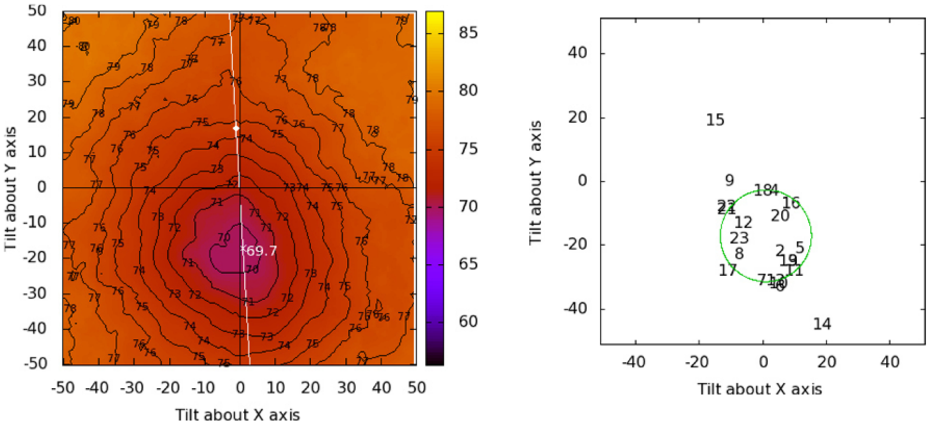

B

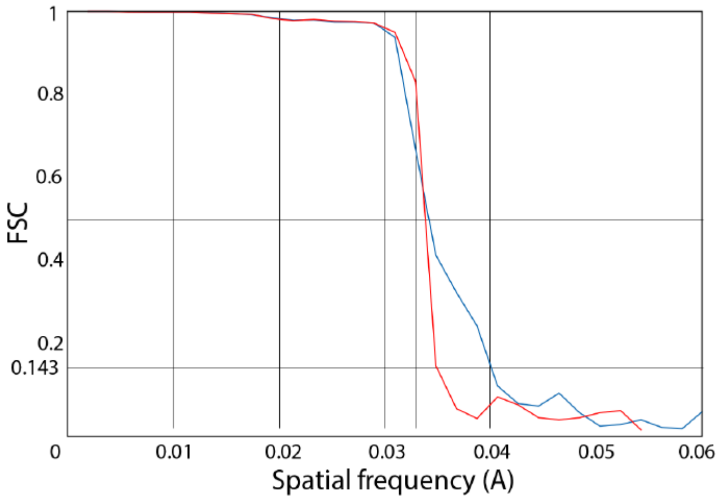

Figure S2 (Related to Figure 2) **EM analysis (A)** (Left) Tilt-pair phase residual plot and (Right) tilt-pair parameter plot shows a minimum and cluster respectively near the tilt-axis and tilt-angle (20 deg) used in the experiment. **(B)** Fourier–Shell Correlation for the crosslinked BRCA1-A complex between two maps calculated from different subsets of particle images (blue) and for refinement performed with phases randomized after 30A (red).

Figure S3

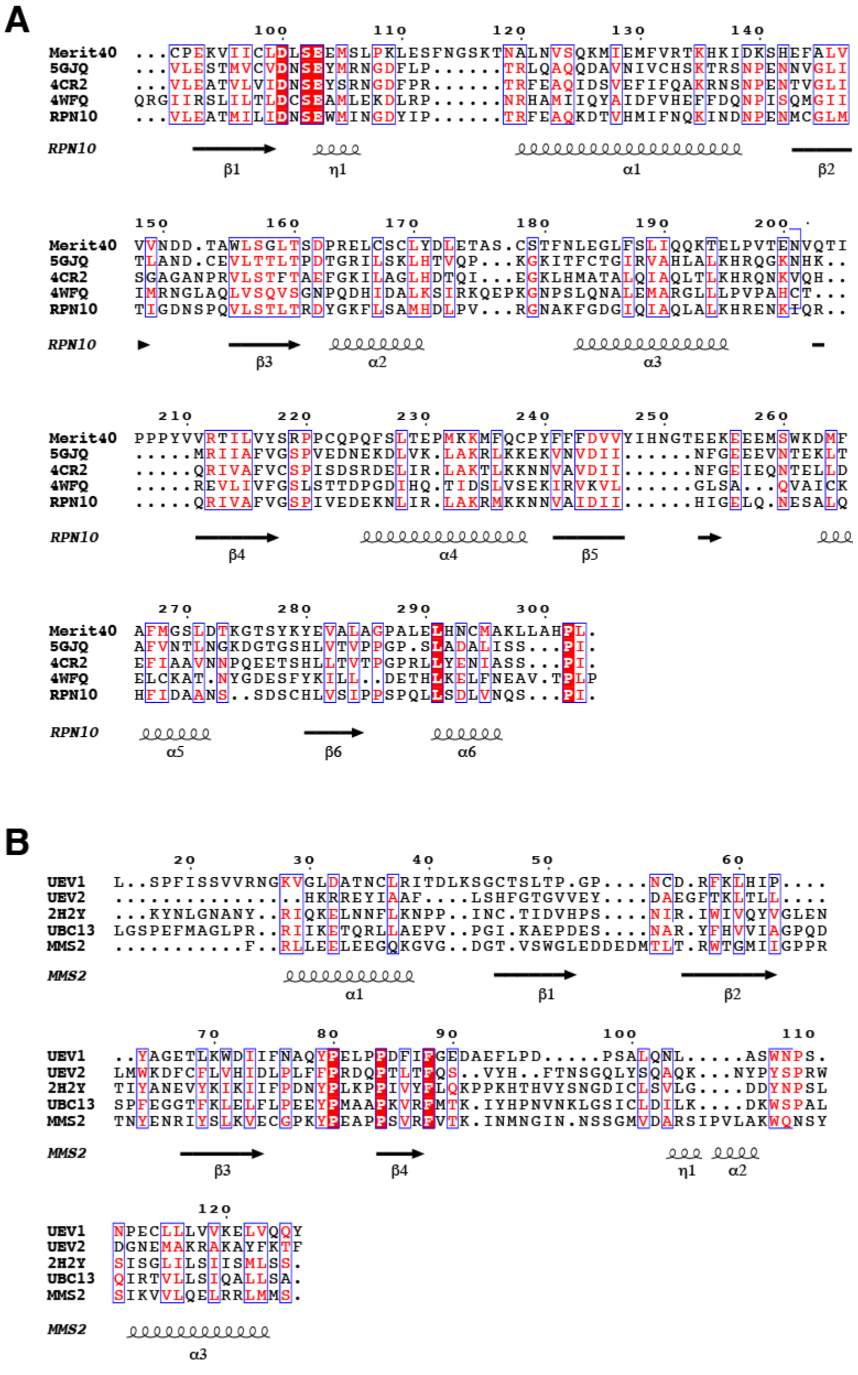

Figure S3 (Related to Figure 3) **Sequence homology** - Sequence alignments of the UEV domains of BRCC45 **(A)** and the VWA domain of MERIT40 **(B)** with homologous domains of known structure.

Supplementary References

Wasilewski S, Rosenthal PB Web server for tilt-pair validation of single particle maps from electron cryomicroscopy. *J Struct Biol* 2014, 186:122-131.

Rosenthal PB, Henderson R Optimal determination of particle orientation, absolute hand, and contrast loss in single-particle electron cryomicroscopy. *J Mol Biol* 2003, 333:721-745.
